# 

**Published:** 1890-06

**Authors:** 


					﻿Vol. X.
JUNE, 1890.
No. £
®HE
JIOMIOPATHIC pHY^lClAJI,
A Monthly Journal of Homoeopathic Materia Medica
and Clinical Medicine.
“ He is a freeman whom truth makes free, and all are slaves beside."
"Seek the Truth: come whence it may, cost what it will."
EDITED AND PUBLISHED BY
WALTER IM. JAMES, IM. E>.,
AND
George H. Clark, im. d.
ASSISTED BY THE FOLLOWING CONTRIBUTORS:
E. T Balch, M. D., South Bend, Wash.
Clarence Willard Butler, M. D.,
Montclair, N. J.
Prof. Edmund Carleton, M. D., New
York.
Daniel W. Clausen, M. D., Philada.
Stuart Close, M.D., Brooklyn.
Edward Cranch, M. D., Erie, Pa.
W. S. Gee, M. D., Hyde Park, Ill.
Clarence C. Howard, M.D., New York.
Samuel a. Kimball, M. D., Boston.
Edmund J. Lee, M. D., Former Editor,
Philada.
A. McNeil, M. D, San Francisco. .
C. F. Nichols. M. D., Boston.
Mahlon Preston, M. D., Norristown.
George W. ShiSrbino, M. D., Abilene,
Texas.
C. Carleton Smith, M. D., Philada.
Wm. Steinkauf, M. D., St. Charles, Mo.
PHILADELPHIA :
No. 1125 SPRUCE STREET.
LONDO :nt
ALFRED HEATH <fc CO., Sole Agents for Great Britain,
114 Ebury Street, Eaton Square.
^Yearly Subscription, $2.50, invariably in advance. Single numbers, 25 cts.
Entered at the Philadelphia Peet-Office as 8econd<ClaM Mail Matter.
				

